# Health impacts of environmental contamination of micro- and nanoplastics: a review

**DOI:** 10.1186/s12199-020-00870-9

**Published:** 2020-07-14

**Authors:** Baorong Jiang, Alexandra E Kauffman, Lei Li, Wayne McFee, Bo Cai, John Weinstein, Jamie R Lead, Saurabh Chatterjee, Geoffrey I Scott, Shuo Xiao

**Affiliations:** 1 Department of Environmental Health Sciences, Arnold School of Public Health, University of South Carolina, Columbia, SC 29208 USA; 2 NIEHS Center for Oceans and Human Health and Climate Change Interactions (OHHC2I), University of South Carolina, Columbia, SC 29208 USA; 3 Center for Global Health, School of Public Health, Nanjing Medical University, Nanjing, 21009 China; 4 National Centers for Coastal Ocean Science, National Oceanic and Atmospheric Administration (NOAA), Charleston, SC 29412 USA; 5 Department of Epidemiology and Biostatistics, Arnold School of Public Health, University of South Carolina, Columbia, SC 29208 USA; 6 Department of Biology, The Citadel, Military College of South Carolina, Charleston, SC 29409 USA; 7 Center for Environmental Nanoscience and Risk, Arnold School of Public Health, University of South Carolina, Columbia, SC 29208 USA; 8 Department of Pharmacology and Toxicology, Ernest Mario School of Pharmacy, Environmental and Occupational Health Sciences Institute, Rutgers University, Piscataway, NJ 08854 USA

**Keywords:** Microplastics, Nanoplastics, Health impacts, Additives, Adsorbents

## Abstract

Plastics are extensively used in our daily life. However, a significant amount of plastic waste is discharged to the environment directly or via improper reuse or recycling. Degradation of plastic waste generates micro- or nano-sized plastic particles that are defined as micro- or nanoplastics (MNPs). Microplastics (MPs) are plastic particles with a diameter less than 5 mm, while nanoplastics (NPs) range in diameter from 1 to 100 or 1000 nm. In the current review, we first briefly summarized the environmental contamination of MNPs and then discussed their health impacts based on existing MNP research. Our review indicates that MNPs can be detected in both marine and terrestrial ecosystems worldwide and be ingested and accumulated by animals along the food chain. Evidence has suggested the harmful health impacts of MNPs on marine and freshwater animals. Recent studies found MPs in human stool samples, suggesting that humans are exposed to MPs through food and/or drinking water. However, the effect of MNPs on human health is scarcely researched. In addition to the MNPs themselves, these tiny plastic particles can release plastic additives and/or adsorb other environmental chemicals, many of which have been shown to exhibit endocrine disrupting and other toxic effects. In summary, we conclude that more studies are necessary to provide a comprehensive understanding of MNP pollution hazards and also provide a basis for the subsequent pollution management and control.

## Introduction

Plastics are synthetic products that are typically made of organic polymers and other chemical additives, such as bisphenols, phthalates, and flame retardants, giving plastic products unique properties [1]. Plastics are used in a variety of commercial applications because of their low cost, ease of production, versatility, and hydrophobicity. The amount of plastics produced is increasing every year; however, the strategies of reusing, recycling, and repurposing have not been implemented accordingly, particularly in some developing countries [2, 3]. It is estimated that about 6.3 billion tons of plastic waste had been generated worldwide from 1950s to 2015 [4]. If this trend continues, that number will increase to 26 billion tons by 2050 [4, 5]. However, only 21–26% of the plastic waste was appropriately recycled and incinerated. The rest is incinerated in open pits or discarded to the environment, leading to plastic pollution of the water, air, soil, etc. [4, 6, 7].

After entering the environment, interactions between the plastic waste and environmental components can degrade large pieces of plastics to smaller plastic debris [8–10]. In addition, tiny plastic particles are commonly manufactured already and added to consumer products such as personal care products that are discarded after use, leading to another important direct source of plastic pollution in the environment [11–13]. According to the diameter of plastic fragments or particles, plastic particles can be divided into microplastics (MPs) and nanoplastics (NPs), with MPs being less than 5 mm in diameter and NPs being 1 to 100 or 1000 nm in diameter [14–19].

Thus far, micro- and nanoplastics (MNPs) have been detected worldwide in both marine and terrestrial ecosystems including oceans, rivers, air, drinking water, sediments, and food [11, 20, 21]. Previous studies have reported that the exposure of MNPs can cause reproductive toxicity in oysters [22], liver toxicity in zebrafish [23], and tissue bioaccumulation and potential organ toxicities in mice [24–27]. These results indicate that the pollution of MNPs is widespread, and the biological harm of MNPs to both humans and other living organisms cannot be ignored. However, the obtained experimental results are not conclusive; the conclusions given by different studies are somewhat conflicting; and the underlying mechanisms of discovered toxicities are still poorly understood. Moreover, recent studies have found MPs in human feces, suggesting that humans are exposed to MNPs through the food chain or food web [28, 29]. Nevertheless, the impact of MNPs on human health has been scarcely researched. Furthermore, in addition to the MNPs themselves, these tiny plastic particles can release plastic additives and/or adsorb other environmental chemicals, many of which have been shown to exhibit endocrine-disrupting and other toxic effects. However, how MNPs will impact the toxicities of these additives and adsorbents is still largely unknown. In this review, we therefore first reviewed the basic properties, sources, and abundance of MNPs in the environment and then discussed the health impacts of pristine MNPs as well as their associated adsorbents and additives.

## Type and use of plastics

A main classification of plastics is based on the durability or non-durability of their shapes, or whether they are thermosets or thermoplastics. Thermosets include polyurethane, epoxy, and alkyd, and they are often used as insulators, adhesives, and plywood*.* The thermosetting process is primarily based on heat-induced crosslinking to form new and irreversible covalent bonds, which makes the thermosets stable and not easy to decompose [30]. On the contrary, thermoplastics have no newly formed chemical bonds and can be recycled and remolded, making them more widely used than thermosets in consumer goods [31–34]. There are four different kinds of thermoplastics: polyethylene (PE), polypropylene (PP), polystyrene (PS), and polyvinyl chloride (PVC). PE is used in a wide variety of inexpensive plastic products, including plastic bags and bottles. There are two commonly used subtypes of PE: (1) the high-density polyethylene (HDPE), which is usually used in detergent bottles, milk cans, and molded plastic cases; and (2) the low-density polyethylene (LDPE) used in outdoor furniture, siding, floor tiles, shower curtains, and clamshell packaging. PP is primarily used to make bottle caps, drinking straws, yogurt containers, appliances, car bumpers, fishing lines, and plastic pressure pipe systems. PS is the primary chemical used to produce foam peanuts, food containers, plastic tableware, disposable cups, plates, cutlery, CD discs, and cassette boxes. PVC is the major component of plumbing pipes and guttering, shower curtains, window frames, and flooring. In addition to the typical plastic classifications listed above, microplastic fibers (MFs), which are made of polyester (PES) or PP, are one of the most common types of MPs found in the environment [35, 36]. MFs are commonly used in a variety of fibrous materials, such as clothing, agricultural, industrial, and household textiles, as well as some textile products, semi-finished or ancillary products used in other fields [37].

Generally, PE, PS, and PVC are three major types of MPs used in scientific research. PE and PS are the most popular plastic materials used in consumer products, and they have shorter service lives than other types of plastics. Additionally, PVC is primarily used for plastic wire insulation or the cable jacket of data cables. Once the life cycle of a cable ends, the metals in the cable will be recycled, but the plastic parts containing PVC are typically discarded into the environment because of the high cost of separation and limited recycling value. It has been reported that 82% of PVC waste is discarded in landfills, 15% is incinerated, and only 3% is recycled [38]. This relationship between the large output, short life cycle, and abundant environmental discharge of these plastics makes them the main focus of scientific research [24, 25, 39–41].

## MNPs in the environment

MNPs, produced or made from a variety of types of plastics, are ubiquitous in the environment. Understanding where they come from and where they go can greatly help study their impacts on environment and human health. It is now generally accepted that MPs are plastic particles with a diameter less than 5 mm [15, 16, 42]. NPs are generally considered to be nano-sized plastic particles, with diameters between 1 and either 100 or 1000 nm [1, 14, 17, 18].

### Sources and formation of MNPs

According to the formation mode, MNPs can be divided into primary MNPs and secondary MNPs [9]. Primary MNPs are processed plastic particles that are commonly added to personal care products [17, 43–46]. These PE microbeads are widely used as exfoliants in cosmetics, detergents, toothpastes, scrub facial cleansers, and drug carriers. Because the primary MNPs added to consumer products mainly serve as physical stimulus and carrier for cleaning; they are easy to be discharged into the environment after use [47]. In addition, a recent study also suggested that glitters that are commonly used in cosmetics, crafts, and textiles are another important source of plastic contamination caused by primary MNPs [48].

**Fig. 1:**
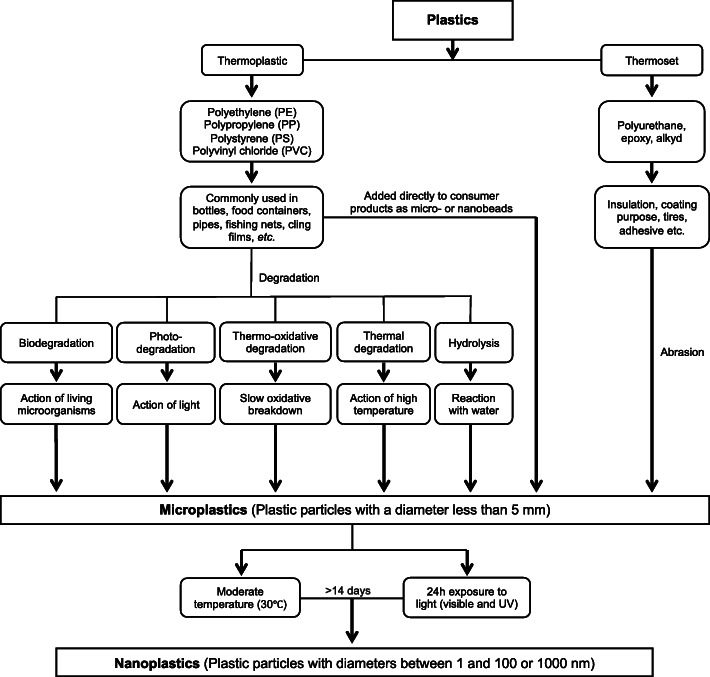
A summary of possible environmental degradation pathway of plastics. This schematic is drawn based on [11, 18]

The second source of MNPs is plastic debris that degrades from the large pieces of plastics due to UV radiation, physical wear, and biodegradation in the environment [11–13, 17, 24, 49]. After plastics enter the environment, they are exposed to UV radiation that catalyzes the photo-oxidation of plastics, making them brittle. Upon further interactions with the wind, waves, and other abrasive interactions, the structural integrity of the plastics further weakens, and MNPs are formed and released from the plastic surface through delamination [9, 11–13, 16, 17, 24, 49–52]. These results indicate that both MPs and NPs can be produced in the degradation process of disposable plastic waste and accumulate over time [18, 53, 54]. Based on these facts, we summarized the pathway of environmental degradation of plastics as well as the production of MPs and NPs in Fig. 1.

### Occurrence of MNPs in the environment

Both MPs and NPs have been found in the marine and terrestrial ecosystems, including oceans, rivers, air, drinking waters, sediments, and foods, worldwide [11, 20, 21, 55]. For example, MPs can be detected in most of the oceans, and they can also be absorbed and bioaccumulated by marine animals. Thus, the study of the health impacts of MPs on animals is an important research direction [55, 56]. In addition, MPs has been found to be present in the soil as well as in earthworms that live on the surface and deep layers of the soil [57–60]. In Table 1, we summarized the literatures focusing on the current environmental pollution of MNPs since 2016, including detection regions, sample sources, abundance, size, and qualitative and quantitative methods. Studies published before 2016 are summarized in another review manuscript [21].

**Table 1:** Summary of published studies providing data on abundance, size range, and qualitative and quantitative methods of micro- and nanoplastics (MNPs) from 2016 to 2020

Region	Sample	Abundance	Size range	Ref
Lake Winnipeg, Canada	Water	193,420 ± 115,567 particles/km^2^	< 5 mm	[61]
Qualitative method: dissecting microscope, scanning electron microscope (SEM), energy dispersive X-ray spectroscopy (EDX)				
Quantitative method: The package “ggmap” and “ggplot2”				
The Hanjiang River and Yangtze River of Wuhan, China	Water	1660.0 ± 639.1 to 8925 ± 1591 n/m^3^	50 μm to 5 mm	[62]
Qualitative method: stereoscopic microscope, SEM, Fourier-transform infrared spectroscopy (FTIR)				
Quantitative method: microscope counting				
Drinking bottled waters, Germany	Water	193 ± 162 particles/l	1 to 500 μm	[63]
Qualitative method: micro-Raman spectroscopy				
Quantitative method: micro-Raman spectroscopy with binary computer				
Drinking water treatment plants, The Czech Republic	Water	1473 ± 34 to 3605 ± 497 particles/l in raw water, 338 ± 76 to 628 ± 28 particles/l in treated water	1 to 10 μm	[64]
Qualitative method: FTIR spectrometer Nicolet 6700, Raman spectroscopy, SEM-EDX				
Quantitative method: VEGA high-resolution SEM				
All steps of drinking water purification, transportation, and supply in drinking water treatment plants (DWTP)	Water	0 to 7 microplastics/m^-3^ (raw water), 0.7 microplastics/m^-3^ (drinking water)	50 to 150 μm	[65]
Qualitative method: micro-FTIR				
Quantitative method: micro-FTIR				
Tyre wear and tear simulator	Tyre wear and tear particles	0.81 kg/year per capita	10 nm to 100 μm	[53, 54]
Qualitative method: particle size analyzer, SEM-EDX, TLM, transmission electron microscopy (TEM)				
Quantitative method: the tyre number weight loss method, the emission factor per vehicle-km approach				
The Changjiang Estuary, China	Sediment	121 ± 9 items/kg of dry weight	1174.5 ± 41.8 μm	[66]
Qualitative method: micro-FTIR				
Quantitative method: micro-FTIR				
Rivers and tidal flat in Shanghai urban districts, China	Sediment	802 ± 594 items/kg of dry weight	100 μm to 5 mm	[67]
Qualitative method: microscopic observations, micro-FTIR				
Quantitative method: weighing method				
The coastline of Tamil Nadu, India	Sediment	46.6 ± 37.2/m^2^	0.3 to 4.75 mm	[68]
Qualitative method: NIKON stereoscopic microscope fitted with a digital camera, Perkin Elmer, attenuated total reflectance (ATR) FTIR (ATR-FTIR)				
Quantitative method: FTIR				
Charleston Harbor, USA.	Water	6.6 ± 1.3 particles/l	> 63 μm	[69]
Qualitative method: ATR-FTIR, dissecting microscope				
Quantitative method: FTIR				
Winyah Bay, USA.	Water	30.8 ± 12.1 particles/l	> 63 μm	[69]
Qualitative method: ATR-FTIR, dissecting microscope				
Quantitative method: FTIR				
Spanish table salt	Salt	50–280 MPs/kg salt	30 μm to 3.5 mm	[70]
Qualitative method: stereo microscopy, FTIR				
Quantitative method: FTIR				
Table salts for sale, China	Sea salts, lake salts, and rock/well salts	550–681 particles/kg (sea salts), 43–364 particles/kg (lake salts), and 7–204 particles/kg (rock/well salts)	< 200 μm	[71]
Qualitative method: Carl Zeiss Discovery V8 stereomicroscope, micro-FTIR				
Quantitative Method: microscopic observation, micro-FTIR				
The Fram Strait, the Barents Sea slope and the Central Arctic	Sea ice	11.7 ± 7.6 N/m^3^	< 50 μm	[72]
Qualitative method: a Hyperion 3000 microscope (Bruker Optics) attached to a Tensor 27 (Bruker Optics) spectrometer, imaging FTIR				
Quantitative method: focal plane array (FPA) FTIR microscopy and image analysis				
Southeastern National Park Service (NPS) units, USA	Sand	43 to 443 pieces/kg sand	~20 μm in width and varied highly (0.1 to 11 cm) in length	[73]
Qualitative method: FTIR				
Quantitative method: microplastic quantification (AM3011 digital microscope)				
Switzerland	Soil	Not mentioned	1–5 mm	[74]
Qualitative method: ATR-FTIR				
Quantitative method: precisely measuring the size of the single particles and calculating their weight using an empirical relationship between particle size and weight				
The central district of Tehran, Iran	Deposited urban dust	Adults: 1063 particles/year; Children: 3223 particles/year	250 to 500 μm	[75]
Qualitative method: SEM, EDX detector				
Quantitative method: binocular microscope				
The North Atlantic subtropical gyre	Seawater	Several populations (13–501 plastic debris per m^3^)	1 to 1000 nm	[18]
Qualitative method: dynamic light scattering (DLS) experiments, FTIR, pyrolysis coupled with gas chromatography-mass spectrometry, microscope imaging				
Quantitative method: principal component analysis (PCA)				

There are some common detection methods for MNPs, including Raman spectroscopy, micro-Fourier-transform infrared spectroscopy (Micro-FTIR), scanning electron microscopy (SEM), transmission electron microscopy (TEM), and energy dispersive X-ray (EDX) [53, 54, 61, 62, 64–73, 75]. Studies focused on the contamination of NPs have just begun, with the first discovery of NPs in the North Atlantic subtropical circulation [18]. The lack of research on NPs is mainly due to the inability of analytical techniques applied to MPs, such as FTIR and Raman spectroscopy, to be used for nano-sized particles. Furthermore, other methods established for characterizing NPs, such as TEM, are unable to clearly distinguish between plastics and other materials such as natural organic matter. Recently, the scanning transmission X-ray microscope (STXM) was used to analyze NPs in soils at a resolution of about 30 nm. However, this experiment only introduced the usability of this method, and the specific results were not given [74]. Additionally, other studies have shown that MNPs can be detected by optical measurement, which provides a basis for later detection methods [76, 77]. Building on the studies investigating the distribution of MPs before 2015 [21], recent studies have increasingly focused on the pathways that allow direct exposure of MPs to humans, such as drinking water and table salt [33, 63, 64, 70, 71]. Taken together, although MNPs have been detected in the biosphere of the sea, land, air ecosystems, and food (chains), at present, there is no unified qualitative and quantitative method to identify their presence or quantify them. Therefore, it is challenging to analyze the real abundance of MNPs accurately in the environment.

## Health impacts of MNPs

In response to the studies suggesting that MPs are ubiquitously present in various environment media, their health impacts on both humans and other organisms have become one of the research foci. Moreover, the trophic transfer of plastic particles may be a common phenomenon that occurs at the same time, making the health impact of MNPs extensive and complex [49]. A recent review summarized the presence of MNPs in animals and foods and elucidated the widespread biological exposure of MNPs [78], suggesting that understanding the health impacts of MNPs is an urgent and unmet need. Here, the health impacts of MNPs themselves, the adsorbents, and the plastic additives are reviewed and discussed.

### Health impacts of pristine MNPs

#### Health impacts of MNPs on marine animals

Since oceans can serve as the ultimate repository for plastic waste, numerous studies focusing on the health impacts of MNPs used non-mammalian marine animals as research models [55, 79]. Furthermore, some of these organisms, such as most of the bivalves, are also used because they are an important food source for humans, representing one pathway by which human may be exposed directly to plastic particles.

Bivalves are a group of animals that lack some of the common molluscan organs, such as the radula and odontophore, so they cannot chew when they eat. All their ingested food goes directly into the digestive system and can be used in MNPs research [80–85]. Most bivalves are filter feeders, including oysters, clams, shellfish, mussels, etc*.* As a result, they eat plastic particles small enough to accumulate in their bodies and cause harmful health effects. Studies have found that plastic particles larger than 4 μm can remain in the body of the blue mussel, and particles smaller than 10 μm can accumulate in the gut and be absorbed into their circulatory system [21, 86]. In addition, another study found that when blue mussel (*Mytilus edulis*) larvae were exposed with the same mass of plastic particles, the intake of 2 μm particles was more than the smaller particles with diameter at 100 nm [87]. The differential intakes of different-sized plastic particles may be due to the fact that the 2-μm particles were mistakenly ingested as food (1–9 μm), while the 100-nm particles float in the water and enter the digestive tract passively with the water. Results from the same study showed that although the growth of blue mussel larvae was not affected, abnormal development increased, and malformation appeared in all treatment groups (0.42 g/L, 28.2 g/L and 282 g/L) of both sizes of plastic particles [87]. Another study found that oyster larvae can generally ingest plastic particles of 160–7.3 μm. Additionally, when oyster *Crassostrea gigas* larvae (3–24 days post fertilization, d.p.f.) were exposed to 1- and 10-μm PS particles for 8 days at the concentrations of 0.11 × 10^-3^ μg/ml and 0.18 g/ml, respectively, there was no measurable adverse effect on the growth, development, or feeding capacity [88]. Another study reported that adult oysters ate PS microspheres and preferred 6 to 2–μm particles at the exposure concentration of 0.023 mg/l [22]. It was postulated that the adult oysters preferred 6- to 2-μm plastic particles because 6-μm particles were more similar in size and shape to their diet [22]. In the same study, MPs was found to significantly reduce the number of follicles and sperm motility in oysters as well as the production and development of offspring larvae after a 2-month maternal exposure experiment [22]. Similarly, another study also reported that exposure to 50 nm NPs can lead to a significant decrease of oyster fertilization rates and embryo–larval development, including many deformities, which results in the complete stagnation of development [89].

MPs have been found to present in the soft tissues of two common bivalves that humans consume: *Mytilus edulis* and *Crassostrea gigas* [39, 84, 90]. Based on the abundance of MPs in the bodies of these two commercial bivalves, European consumers of shellfish are estimated to intake 11,000 kinds of MPs in their diet each year, indicating that the MPs accumulated in bivalves could be an important exposure route for people who consume seafood [84]. Among different sources of clams (*Venerupis philippinarum*), studies found that there was no significant difference for the intake and accumulation of MNPs between wild and farmed clams, with the concentration of detected plastics ranging from 0.07 to 5.47 particles/g [91]. Moreover, researchers found that the characteristics of MPs in clams are similar to those in sediments, suggesting that clams can be used as a biological indicator of microplastic pollution in sediments [92].

There are also studies looking at the possible biological changes caused by plastic particles in other marine animals. For instance, lugworms (deposit feeders) are large marine worms of the phylum Annelida. In the natural ecological environment of the coastline of northern Europe, there were 1.2 ± 2.8 particles/g in the lugworms (*Arenicola marina*) [39]. However, results also showed that these accumulated plastic particles did not have significant effects on the organisms, nor did they enhance or weaken the bioaccumulation of other chemicals [93]. The results from another study using *Dunaliella salina* indicated that 200 μm MPs, which are larger than a cell, promoted *D. salina*’s growth and photosynthesis at the concentrations of 200, 250, 300, and 350 mg/l and that the adverse effects increased as the diameter of MPs decreased. These results suggested that the size of MPs is closely related to the corresponding biological effects, and that NPs may cause more serious biological toxicity than MPs [94]. Regarding marine invertebrates, studies reported that larval and juvenile *Crepidula onyx* grew slowly after exposure to relatively high concentrations (1.4 × 10^5^ particles/ml) of micro-PS, suggesting that MP exposure may cause abnormal energy consumption [95].

The results described above indicate that MNPs of different sizes can be differentially absorbed and accumulated by marine organisms, and the ingested plastic particles have various health impacts on different marine species. Most marine filter feeders prefer to ingest MNPs less than 10 μm, and NPs are smaller than MPs and more likely to be ingested, leading to higher concentrations in the body and greater toxicity in circulation. In addition to the size-dependent effects, there is also dose-dependent effect of MNPs. The toxicity of any substance is determined by its concentration and diameter and other physical parameters. Therefore, the size of the MNPs and the high and low concentrations will cause inconsistency in the toxic effects caused by MNPs. More sophisticated and targeted evaluations are necessary to determine the health impacts of MNPs on marine animals.

#### Health impacts of MNPs on freshwater organisms

The impact of MNPs on organisms found in freshwater habitats has also attracted extensive attention. Studies have shown that different concentrations of MNPs in the terrestrial aquatic ecosystems have various degrees of influences on the growth, development, behavior, reproduction, and mortality of aquatic animals (represented by *Daphnia* and zebrafish) [15].

*Daphnia magna* (*D. magna*) is a small planktonic crustacean with adult length at 1.5–5.0 mm. They are widely used in aquaculture and aquaria as fish food and have been used as one of the biological research subjects since the 18th century. More importantly, *D. magna* is used in the OECD Guidelines for the Testing of Chemicals in ecotoxicology. In recent years, studies about the effects of plastic fragments on aquatic organisms using *D. magna* have focused on the bioaccumulation of plastic particles in their intestinal tissues, survival rate after exposure, and possible reproductive toxicity. In one study with exposure to four types of environmentally relevant MPs at the concentration of 100 mg/l for 48 h, MPs were found in the gut of *D. magna*, but no acute effects were observed [96]. Additionally, after a short-term exposure of 12.5–400 mg/l with diameters of 1 μm and 100 μm PE MPs for 96 h, Rehse and colleagues found that the effect of 1-μm plastic particles on *D. magna* immobilization changed in a time- and dose-dependent manner. However, the 100-μm-sized plastic particles could not be ingested, and there was also no significant harmful effect for this size of plastic particles [97]. Another study used 100 nm and 2 μm fluorescent PS MPs to investigate the effect of MPs on the feeding and reproduction rate of *D. magna*. This study was divided into two parts. First, the animals were exposed to 1 mg/l MPs for 24 h and were then purified for 24 h to assess the intake of MPs in the animals. The second part was a 21-day exposure of 0.1 mg/l, 0.5 mg/l, and 1 mg/l MP, respectively, to determine the toxic effect of MPs on the reproduction of *D. magna*. Their results showed that both of sizes of MPs were easy to ingest, and the intake of 2-μm particles was five times higher than the 100-nm particles. After 21 days of exposure to 0.1 mg/l, 0.5 mg/l, and 1 mg/l MPs, there was no observed adverse effect on reproduction. However, the exposure of 100-nm MPs resulted in a reduction in the excretion rate and feeding rate of *D. magna*, indicating that plastic particles in the nanoscale were more harmful to *D. magna*. than those in the microscale [98]. This may be because NPs are small in diameter and are more likely to remain in the digestive tract, where physical accumulation leads to a false sense of fullness, prompting organisms to eat less. Other studies have reported no increase in adult *D. magna* mortality after MP exposure, no change in morphology (length, width, and tailbone length), and no harmful effect on reproductive parameters. Taken together, these results suggest that the ingested MNPs can pass through the gut of *D. magna*; however, whether the absorbed MNPs will result in adverse health impacts on *D. magna* requires further investigation [96–100].

Zebrafish (*Danio rerio*) is a type of ornamental freshwater fish and has been widely used as a vertebrate model in toxicological research. The absorption and accumulation patterns of zebrafish has been examined using a 7-day exposure to 5 μm and 20 μm of PS MPs at the concentrations of 20 mg/l. In the same study, the liver toxicity was also investigated using a 3-day exposure of 70-nm and 5-μm PS MPs at the concentrations of 20, 200, and 2000 mg/l. The results showed that 5-μm PS MPs can accumulate in the gills, liver, and gut, but 20-μm PS MPs could not accumulate in the gill tissue. The results of organ toxicity assessment indicated that both 70-nm and 5-μm PS MPs can induce inflammation and lipid accumulation in the liver. Meanwhile, changes of oxidative stress and lipid energy metabolism were noted by analyzing the increase or decrease of some enzyme activities [23]. Another study has shown that MPs do not cause or rarely cause death in zebrafish (*Danio rerio*) after a 10-day exposure to 0.001–10.0 mg/l MPs. However, intestinal damages, including cracking of villi and splitting of enterocytes, were noted after exposure to all four common MPs, including polyamides (PA), PE, PP, and PVC [101]. The exposure of NPs has been found to result in developmental toxicity in zebrafish. When zebrafish embryos were exposed to 0.1, 1, or 10 ppm of PS NPs 24 h post-fertilization (hpf) with an average diameter of 51 nm, the NPs were found to accumulate in the yolk sacs and migrate to the gastrointestinal tract, gallbladder, liver, pancreas, heart, and brain at 48–120 hpf. The accumulation of PS NPs decreased during the purification period of all organs (120–168 hpf), but the clearance rate of the pancreas and gastrointestinal tract was slower than other organs. Notably, exposure to PS NPs did not result in significant mortality, malformation, or mitochondrial bioenergy changes, but reduced the heart rate of zebrafish embryos. In conclusion, these data suggest that NPs can penetrate the choroid membranes of developing zebrafish, accumulate in embryonic tissues, and influence physiology and behavior, leading to inter- or transgenerational toxicity [102, 103].

In addition to using the commercially uniform MNPs, the isolated MNPs from environmental samples is a more realistic and representative approach to perform the exposure experiments. A recent study found that a 21-day exposure of MFs increased oviposition and secondary patellar aneurysms in adult Japanese medaka (*Oryzias latipes*) at the concentration of 10,000 particles/l [35]. This study provides some insight that MNPs obtained from environmental samples can be directly used in toxicological assessments. Compared with the commercially produced plastic microspheres, the natural MNPs with environmental separation has more research value and exploration significance.

#### Effects of MNPs on mammalian species

Recently, researchers have begun to use mammalian animal models to predict the potentially harmful impact of MNPs on human health. It has been found that mice exposed to PS MPs with diameters at 5 and 20 μm for 28 days showed the presence of MPs in the liver, kidney, and gut [24]. Moreover, the results related to energy, lipid metabolism, etc. suggested the possible harmful effects after exposure to MPs. For example, the levels of T-CHO and TG were significantly reduced in the MP-treated group, and lipid droplets were detected in the liver, suggesting that MPs can cause lipid metabolism disorders and liver inflammation in mice. Another two studies have shown that mice exposed to PS MPs had decreased intestinal mucus and significant changes in the richness and diversity of intestinal biota [26, 27]. Regarding NPs, Rafiee et al. analyzed the effect of long-term exposure of PS NPs on neural behavior in rats. Specifically, adult male Wistar rats were exposed to 1-, 3-, 6-, and 10-mg PS NPs/kg of body weight/day. The particles had an average diameter of 38.92 nm and were exposed orally for 5 weeks. Results indicated that no significant behavioral effects were noted in all neurobehavioral tests. However, some subtle toxic effects, such as decreased locomotor activity, were observed, which provides insight for future studies [104].

Based on the results obtained from mammalian animal models, it is reasonable to assume that plastic particles can possibly accumulate and affect human health. According to the results in Table 1, the average abundance of MNPs in drinking water is 193 particles/l, which is much lower than the concentration used in the cited studies above. However, based on the fact that people drink 1200–1600 ml water per day, it may be important to consider the long-term exposure to MNPs. Additionally, MNPs have also been detected in table salt, honey, and sugar, indicating other sources of MNP exposure to humans [21]. However, there are no accurate data to determine the daily exposure and intake of MNPs. It is also not conclusive that whether the MNPs absorbed by the human body enter the internal circulation through gastrointestinal tract and ultimately cause organ damage. Therefore, more exposure and toxicity assessments using human relevant experimental models are necessary.

### Effects of adsorbents of MNPs

Current studies suggest that hazardous chemicals can be adsorbed onto MNPs, and these adsorbed pollutants on MNPs could be many orders of magnitude higher in concentration than those detected in the surrounding environment [40]. Moreover, new studies support the possibility that the adsorbed chemicals exhibit more toxicity than pure chemical alone [40]. Here, we used hydrophobic organic chemicals (HOCs) and heavy metals as examples to review the toxicity of the chemicals involved, their ability to adsorb onto MNPs, and their toxicity when together. Since the current evidence on NPs is limited, we primarily focused on the adsorption of environmental chemicals on MPs.

HOCs exist in many varieties in the environment, and many of them are known to be endocrine-disrupting chemicals (EDCs), such as the PCBs (polychlorinated biphenyls), PFCs (perfluorinated chemicals), BPA (bisphenol-A), and phthalates [105]. HOCs have been shown to adsorb onto MPs. For example, one study measured the partition coefficients between MPs and seawater for various types of HOCs [106]. These results revealed a high sorption capacity of MPs over the aqueous phase, suggesting that MPs can enhance the environmental exposure as well as the corresponding toxicities when coexisting with HOCs. Similar to what we have discussed above, these results are speculated to be a result of the addition of MPs, which, when ingested, facilitated the transfer of PCBs to the organisms.

In terms of heavy metals, MPs can sorb metals from both aquatic and sedimentary environments, allowing for an accumulation of these metals [107]. Additionally, a 12-month study from Rochman et al. demonstrated an increased pattern of several types of heavy metals over time, including cadmium, nickel, zinc, and lead. This suggests that the longer MPs stay at the sea, the more metals they can accumulate. Thus, a mixture of metals, including those listed as priority pollutants by the US EPA, can be found on plastic debris [108]. Heavy metals are a potential hazard to both wildlife animals and humans. For example, mercury has been observed to bioaccumulate alongside MPs in *Dicentrarchus labrax*, the sea bass, and the analysis of mercury detected in the brain and muscle tissues found significant interactions between mercury and MPs [109].

These studies raise concern for the possibility that the body can uptake not only the MPs, but also the adsorbed toxic chemicals that MPs carry. There is also the possibility that a combination of MPs and their adsorbed chemicals can be more toxic than either counterpart on its own. A study examined the exposure to both MPs and organophosphorus flame retardants (OPFRs), which are a type of HOC. The results demonstrated that the co-exposure of these particles induced greater oxidative stress, neurotoxicity, and metabolic disruption in mice than either the MPs or OPFR alone [40]. This could be simply due to an increase in uptake of the toxic chemicals in the presence of MPs. Another possible reason may be that the sorption and desorption equilibrium of the HOC and MPs could slow down the metabolism in mice, causing higher toxicity [40] or, quite simply, it could be explained by the separate toxicity of the MPs and the HOCs, which were both still ingested. These results make MPs even more complex to understand because examining pure plastics in the lab and their impact does not address how plastics adsorb chemicals. Recent studies have also found that PS NPs and natural acidic organic polymers (NAOP) such as fulvic acid and humic acid are jointly exposed to *Scenedesmus obliquus* and zebrafish (*Danio rerio*), which can cause low growth inhibition toxicity of algae, conditioned oxidative stress of cells and impaired mitochondrial function, as well as significant effects on oxidative stress and enzymatic antioxidant defense of zebrafish [110]. The temperature of the environment, mode of transport, and type and size of plastic accumulated can potentially affect which chemicals adsorb, to what degree, and how they will encounter humans or aquatic life. Therefore, further studies are also needed to explore how long these chemicals stay adsorbed to the MPs. Previously, results obtained based on animal experiments have shown that MPs can damage the intestinal barrier and reduce intestinal microbial diversity, so the biohazard of combined exposure deserves attention. Equally important, the biofilm formed on the surface of MNPs may promote biological diffusion, invasion, and mutation. The adhesion of different microbial communities to the MNP surface may enhance the flow rates of energy, material, and information in the environment, with long-term and widespread harmful effects [111].

### Health impacts of plastic additives of MNPs

Another critical issue to be considered is the leaching of original plastic additives, such as BPA, phthalates, OFPRs, etc., which have been demonstrated to exhibit endocrine-disrupting effects and other toxicities [112, 113]. The release of plastic additives may take place during the service life of the plastics or after their disposal. An additive’s migration potential depends on the polymer’s pore diameter, the size of the additive, and its partition coefficient once it reaches the plastic’s surface. For example, the additives of a lower molecular weight move much more easily through a polymer with a bigger pore size [114]. More significantly, the environment of the plastics can affect chemical properties of polymers and their additives. For example, rising temperatures can promote movement of additives in polymers [115], and exposure to UV radiation can increase the rate of plastic degradation [116]. In addition, it has also been demonstrated that plastics that are exposed to the salinity of the water in the ocean can desorb estrogenic plasticizers [116]. Plastics in landfills are exposed to leachates of various acidities and chemical properties. Based on those properties, the leachates have various potentials to extract and transport. Different biological populations also have the potential to degrade or transform the released additives [114]. For instance, bacterial populations can colonize, modify, and degrade MPs [117]. With the knowledge that bacteria are present within organisms, it may be important to consider the role they may play in the release of the additives from ingested MPs and the subsequent ramifications this may have on the exposed organism. Taken together, although studies have shown how the chemistry of the environment or the presence of bacteria can impact plastic degradation, there is little knowledge about how these factors come into play for the organisms who ingest MPs. The extent to which plastics can degrade and release additives within the organisms is unknown. More studies are needed to determine the ability for plastic additives to leach from MNPs to the organisms who ingested them, especially considering the extensive knowledge of the endocrine-disrupting effects and other organ toxicities of these plastic additives.

## Feasible control or disposal measures of MNPs

Since evidence reveals the widespread contamination of MNPs as well as their potentially adverse health impacts on both wildlife animals and humans, researchers also focused on the reduction or elimination of MNPs in the environment, providing possible solutions for protecting both environment and health. One potential option is to look for organisms or other substances that can degrade MNPs. Marine fungus *Zalerion maritimum* and mixed bacteria have been found to have positive and effective effects on plastic degradation [118, 119]. In addition, a recent study reported that PET polyester plastics could be rapidly and efficiently degraded by special enzymes. ICCG and WCCG, two new variants of leaf–branch compost cutinase (LCC), obtained 90% depolymerization in 9.3 h and 10.5 h, respectively. This research develops enzymatic treatment to help solve the plastic treatment problem, which is a good indicator and promotion for achieving sustainable development and circular economy [120]. Furthermore, some traditional plastics have been replaced by biodegradable plastics and degraded by soil composting to reduce environmental pollution. However, these biodegradable plastics also produce MNPs in the composting process, which is worthy of further assessment [121]. For cleaning MNPs in indoor air, the cytotoxic micro polyacrylate styrene and nano-Fe3O4 particles produced by printer toner can be controlled by vacuum-gasification-condensation [122]. Some studies have summarized the pollution situation of MNPs in some developing countries with high population density and fast economic growth, such as India and China, and made useful explorations. For example, the control and elimination of MNPs is advocated to include sewage treatment process to reduce their environmental emissions [3, 123].

## Conclusion

**Fig. 2:**
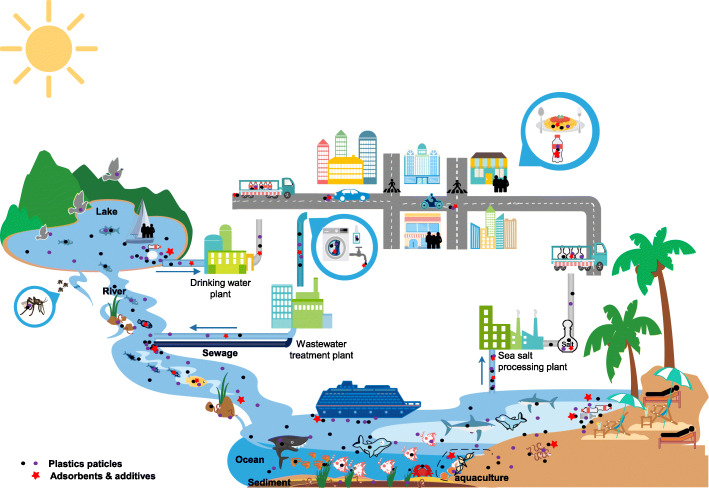
A summary of sources, transports, and exposure routes of micro- and nanoplastics (MNPs)

In Fig. 2, we summarized the sources, transports, deposition, bioaccumulation, and biomagnification of MNPs, as well as the possible exposure routes of MNPs in both humans and other living organisms. Evidence has suggested harmful health impacts of MNPs on marine and freshwater animals. Thus far, there is no conclusive data regarding the health impact of MNPs on humans. However, it is conceivable that MNPs can be slowly eroded into the intestinal wall into the circulatory system and distribute to various tissues and organs. If accumulated over time, their toxic effects may cause corresponding damages to the human body. The research focused on NPs have just begun. Because of the unique size- and shape-dependent properties of nanoparticles, NPs may exhibit significantly differential impacts from MPs. In Table 2, we summarized the health impact of MNPs, including species, plastic size, type of MNP, discovered health effect, and corresponding references. In summary, we conclude that more studies are necessary, including standardizing sampling methods, establishing qualitative and quantitative measures for testing both MPs, NPs, etc. Once these are established, multiple experimental models can be used to study the health impacts of the MNPs themselves, the associated adsorbents and additives on MNPs, as well as the potential biological amplification of the mixtures of these substances.

**Table 2:** A summary of the health impacts of MNPs reviewed in our study

Species	Size	Type	Effects	Reference
Blue mussel	4–10 μm	MP	Remain in the body	[21]
	2 μm, 100 nm	MP, NP	Abnormal development and deformity were found in both MNP treatment groups, but the growth of mussel larvae was not affected.	[87]
Oyster	160 nm –7.3 μm	MP, NP	No measurable adverse effect on the growth, development, or feeding capacity	[88]
	1 μm, 10 μm	MP		
	2 μm, 6 μm	MP	Significantly reduce the number of follicles and sperm motility in oysters as well as the production and development of offspring larvae	[22]
	50 nm	NP	Significant decrease of oyster fertilization rates and embryo–larval development, including many deformities, which results in the complete stagnation of development	[89]
Clam	1.2 μm–5 mm	MNP	No significant difference for the intake and accumulation of MNPs between wild and farmed clams	[91]
Lugworms	10–180 μm	MP	Accumulated plastic particles did not have significant effects on the organisms, nor did they enhance or weaken the bioaccumulation of other chemicals	[93]
	200 μm	MP	Growth and photosynthesis were promoted, and the smaller the particle size was, the more obvious the effect was.	[94]
Crepidula onyx	2.0–2.4 mm	MP	Cause abnormal energy consumption	[95]
Daphnia	20–250 mm	MP	Remains in the gut, but there are no acute effects that can be observed	[96]
	1 μm, 100 μm	MP	The effect of 1-μm plastic particles on immobilization changed in a time- and dose-dependent manner. However, the 100-μm sized plastic particles could not be ingested, and there was also no significant harmful effect for this size of plastic particles.	[97]
	100 nm, 2 μm	MP, NP	The plastic particles of both sizes are easy to ingest, and the uptake of 2-μm particles is 5 times that of 100-nm particles. NP resulted in reduced excretion and ingestion rates, but no adverse effects of MP and NP on reproduction were observed.	[98]
	63–75 μm	MP	No increase in adult *D. magna* mortality after MP exposure, no change in morphology (length, width, and tailbone length), and no harmful effect on reproductive parameters	[96–99, 124]
Zebrafish	70 nm, 5 μm, 20 μm	MP, NP	5-μm MPs can accumulate in the gills, liver, and gut, but 20-μm MPs could not accumulate in gill tissue. In addition, both 70-nm and 5-μm MPs can induce inflammation and lipid accumulation in the liver, with changes in oxidative stress and lipid energy metabolism	[23]
	~70 μm, 0.1 μm, 1.0 μm, 5.0 μm	MP	Causes intestinal damages, including cracking of villi and splitting of enterocytes, but does not or rarely cause zebrafish death. The 1.0-μm particles were highly lethal, had the highest accumulation, the lowest intestinal Ca^2+^ level, and the highest expression of glutathione S-transferase 4	[101]
	20–100 nm	NP	Penetrate the choroid membranes of developing zebrafish, accumulate in embryonic tissues, and influence physiology and behavior, leading to inter- or transgenerational toxicity	[102, 103]
Japanese medaka	50–60 μm	MF	Increased oviposition and secondary patellar aneurysms	[35]
Mice	5 μm, 20 μm	MP	Remain in the liver, kidney, and gut; energy and lipid metabolism disorders and liver inflammation	[24]
	5 μm, 0.5 μm, 50 μm	MP	Decreased intestinal mucus and significant changes in the richness and diversity of intestinal biota	[26, 27]
	38.92 nm	NP	No significant behavioral effects were noted in all neurobehavioral tests. However, some subtle toxic effects, such as decreased locomotor activity, were observed, which provides insight for future studies.	[104]
Human	50–500 μm	MP	Various MPs have been detected in human feces, suggesting that MPs can enter the body through the digestive system and be excreted in feces.	[125]

## Data Availability

All data described, analyzed, or discussed in this review are included in cited publications.

## Abbreviations

MPs: Microplastics
NPs: Nanoplastics
MNPs: Micro- and nanoplastics
PE: Polyethylene
PP: Polypropylene
PS: Polystyrene
PVC: Polyvinyl chloride
HDPE: High-density polyethylene
LDPE: Low-density polyethylene
MFs: Microplastic fibers
PES: Polyester
Micro-FTIR: Micro-Fourier-transform infrared spectroscopy
SEM: Scanning electron microscope
TEM: Transmission electron microscopy
EDX: Energy dispersive X-ray
STXM: Scanning transmission X-ray microscope
*D. magna*: *Daphnia magna*
HOCs: Hydrophobic organic chemicals
EDCs: Endocrine-disrupting chemicals
PCBs: Polychlorinated biphenyls
PFCs: Perfluorinated chemicals
BPA: Bisphenol-A
OPFRs: Organophosphorus flame retardants
NAOP: Natural organic polymers
LCC: Leaf–branch compost cutinase
